# Fabrication of Microneedle Patches by Suspension Casting of Drugs in Organic Solvents

**DOI:** 10.3390/pharmaceutics18060692

**Published:** 2026-06-01

**Authors:** Chao-Yi Lu, Lara Vaid, Asha Adler, Gulcin Arslan Azizoglu, Andrey V. Romanyuk, Mark R. Prausnitz

**Affiliations:** 1 Wallace H. Coulter Department of Biomedical Engineering at Georgia Tech and Emory University, Georgia Institute of Technology, Atlanta, GA 30332, USA; 2 School of Chemistry and Biochemistry, Georgia Institute of Technology, Atlanta, GA 30332, USA; lavaid28@gmail.com; 3 School of Chemical and Biomolecular Engineering, Georgia Institute of Technology, Atlanta, GA 30332, USA; gazizoglu3@gatech.edu (G.A.A.); andrey.romanyuk@chbe.gatech.edu (A.V.R.)

**Keywords:** dissolving microneedle patch, drug suspension, organic solvent casting, microfabrication, transdermal drug delivery

## Abstract

**Background/Objectives:** Drug administration by microneedle patch (MNP) offers advantages over conventional dosage forms as a painless, self-administered skin patch for parenteral delivery. Dissolvable MNPs are typically manufactured by casting an aqueous formulation containing dissolved active pharmaceutical ingredient (API) and excipients into a mold and allowing it to dry. This process can be detrimental to APIs that are sensitive to dissolution and drying during the casting process. **Methods**: This study presents a MNP fabrication process in which drug particles are suspended in an organic solvent carrier without being dissolved in the solvent. **Results**: We started with drug particles either as pure API or formulated with excipients to stabilize them. We then screened nine organic solvents, ranging from high (methanol) to low (toluene) polarity, to identify those that suspend the drug particles without dissolution or damage to the API. To guide formulation of stabilized drug particles, we generated a companion database of 16 common stabilizing excipients and measured their solubility in our panel of organic solvents to identify excipient–solvent combinations that did not lead to excipient dissolution. We generated a second database of 14 water-soluble polymers to serve as the microneedle matrix material and determined their solubility in our panel of solvents to identify solvents that enabled polymer dissolution. Using these data, we designed casting solutions that suspended particles of API (and excipients) in an organic solvent that dissolved a matrix polymer. Casting and drying these solutions on molds produced MNPs for delivery of three model compounds: lyophilized tetanus toxoid (i.e., a vaccine), methotrexate (i.e., a small molecule drug), and insulin (i.e., a biologic). **Conclusions:** We conclude that this fabrication method, guided by the excipient and polymer solubility databases, offers a novel method to produce MNPs by suspension casting of drugs in organic solvents.

## 1. Introduction

Drugs are typically administered orally or through injections. However, oral administration may lead to poor drug bioavailability or absorption through the gastrointestinal tract due to physiological barriers, including enzymes, acidic environment in the stomach, and poor absorption across the mucosal layer [1,2,3]. In addition, some people have difficulty swallowing oral dosage forms, including young children, unconscious people, and those with certain mental and physical disabilities [4,5,6]. Injections can overcome these limitations; however, injections have lower patient acceptability and adherence than oral administration and can lead to needlestick injuries [7]. Injections are typically performed by trained healthcare professionals, making the procedure less accessible to patients. Additionally, the procedure can be painful, leading to needle phobia among patients and lower patient adherence [8,9]. Injections can also cause needlestick injury, which is a common source for transmission of pathogens, including hepatitis B, hepatitis C, and human immunodeficiency viruses [10].

An alternative to these conventional delivery routes is microneedle patches (MNPs), which contain an array of needles that are typically 0.1–1 mm in length and can painlessly penetrate across the outermost layer of the skin, the stratum corneum, which is 10–20 µm in thickness [11,12,13,14]. MNPs can deliver drugs to the skin for local effect or for systemic uptake via the vasculature [15,16]. Compared to conventional drug delivery methods (i.e., oral administration and injections), MNPs avoid poor drug absorption through the gastrointestinal tract and the need for trained healthcare professionals for injections [15,16]. Studies have shown that MNPs can be self-administered and are preferred over injections [15,16]. Furthermore, dissolving MNPs have been developed so that the microneedle matrix dissolves upon insertion into the skin, leaving no biohazardous sharps waste after use and minimizing needlestick injury risks [11].

Dissolving MNPs can be fabricated by a number of different methods, including lithography, droplet-born air blowing, photopolymerization, and micromolding [17,18,19,20]. Each of these methods involves converting a liquid formulation containing an active pharmaceutical ingredient (API) and excipients into solid microneedles, where solidification occurs by drying a solution with heat and/or vacuum, undergoing a chemical reaction, or solidifying a melt by cooling. A limitation of these approaches is that the API can be damaged by the removal of water during drying, ultraviolet light or other conditions during chemical reaction, or heat during melt formation [14,20].

The most commonly used MNP fabrication method is micromolding, during which an aqueous formulation containing API and water-soluble polymer and/or other excipients is cast into the cavities of a poly(dimethyl siloxane) (PDMS) female mold [14,17]. Vacuum or centrifugation is often used during casting to drive the formulation into the mold cavities [17]. Further drying is then performed, and the MNP is then peeled out of the mold [11,17].

APIs are usually cast into solutions because more than 80% of drugs are solids [21]. In other pharmaceutical manufacturing processes, drying of API solutions is often done by lyophilization, which uses freezing and sublimation to remove water while the API is in a frozen state with limited conformational mobility that prevents denaturation or other damage to the API [22]. Drying during MNP fabrication, however, is generally not compatible with lyophilization, because lyophilization produces a porous powder [23] that is typically not strong enough to withstand the forces of microneedle insertion into skin [24]. Micromolding MNPs therefore usually uses air drying instead, which requires optimized formulation and fabrication conditions to preserve API stability. This optimization requires balancing many constraints in addition to API stabilization, including achieving sufficient microneedle mechanical strength, casting solution processability, excipient biocompatibility and other factors. Therefore, it would be advantageous to introduce API as a dry, stabilized powder into the MNP fabrication process without the additional stresses caused by exposure to water and drying.

In previous work, we accomplished this by suspending API particles in a non-solvent, thereby enabling liquid casting to fill the MNP mold with undissolved drug particles. More specifically, we cast lyophilized protein suspended in chloroform with dissolved polyvinylpyrrolidone (PVP) to form the microneedle matrix [24]. Building on this proof-of-principle, we next sought to develop a framework for the rational design of formulations that include (i) a solvent that does not dissolve the API of interest during manufacturing and is applicable to various types of APIs, including small molecules and biologics, and (ii) a water-soluble polymer that dissolves in the solvent and forms the microneedle matrix upon drying.

Specifically, we identified organic solvents from a library of nine solvents spanning a broad range of polarity that can suspend an API of interest and enable retention of drug potency after suspension casting. We also characterized the solubility of 16 stabilizing excipients for possible coformulation with the APIs. We then identified polymers from a library of 14 biocompatible polymers that can be used to form the microneedle matrix and can be dissolved in the organic solvent. Using the resulting combinations of organic solvents and polymers that can suspend the API and form MNPs after drying, we fabricated dissolving MNPs without the need for dissolving the API. We were able to apply this approach to a variety of APIs, including lyophilized tetanus toxoid (TT) vaccine, methotrexate (MTX), and insulin. Through the formulation and fabrication of these MNPs, we present a streamlined method to identify combinations of solvent, polymer, excipient, and API that can be used to develop suspension-cast MNPs for APIs broadly.

## 2. Methods

### 2.1. APIs

APIs used in this study included a lyophilized vaccine, a small molecule drug, and a biologic: lyophilized TT, MTX (Sigma-Aldrich, St. Louis, MO, USA), and insulin (Santa Cruz Biotechnology, Dallas, TX, USA). TT was kindly provided by Serum Institute of India (Pune, India) and lyophilized using the following method.

### 2.2. TT Lyophilization

To prepare lyophilized cakes containing ~50 Lf TT, 5 mg sucrose (Sigma-Aldrich) was dissolved in 85.6 µL deionized (DI) water in a 1.5 mL HPLC vial (Agilent Technologies, Santa Clara, CA, USA). Sucrose was used as the stabilizing excipient due to its ability to retain TT activity after lyophilization (Figure S1a in Supplementary Information). Then, 14.4 µL TT (3480 Lf/mL) was added to the solution. To prepare lyophilized cakes containing ~25 Lf TT, 5 mg sucrose was dissolved in 92.8 µL DI water, and 7.2 µL TT (3480 LF/mL) was added. The cap of the vial was replaced by a piece of folded tissue paper (Kimwipe, Kimberly Clark, Irving, TX, USA) that was taped down to cover the opening of the vial, and the vial was then transferred to a −80 °C freezer for at least 1 h. The shelf of the lyophilizer (VirTis, SP Scientific, Warminster, PA, USA) was set at and stabilized at −60 °C. Before transferring the frozen solution from the freezer to the lyophilizer, the condenser of the lyophilizer was set at and stabilized at −70 °C. After setting the vials in the lyophilizer, the vacuum was then turned on so that the pressure inside the lyophilizer reached 50 mTorr. After ~40 h, the samples were removed from the lyophilizer and covered using the original HPLC caps. Samples were stored at −20 °C until further analysis or use.

### 2.3. API Suspension Test

To identify organic solvents that did not dissolve an API, each API (lyophilized TT, MTX, insulin) was suspended in each organic solvent (methanol, ethanol, 2-propanol, acetonitrile (ACN), acetone, chloroform, ethyl acetate, dioxane (Avantor, Radnor, PA, USA), toluene) in glass vials (Clear Borosilicate Vial, Qorpak, Bridgeville, PA, USA). All solvents other than dioxane were obtained from Sigma-Aldrich. For lyophilized TT, the lyophilized cake containing ~50 Lf TT and 5 mg sucrose was homogenized for 8 s in 1 mL of organic solvent. We used a homogenizer because sonication was detrimental to TT potency (Figure S1b in Supplementary Information). For MTX and insulin, the suspension was prepared at ≥1 mg/mL and vortexed. These vials were then placed on a shaker (Titramax 1000, Heidolph, Schwabach, Germany) at ~400 rpm at room temperature (20–25 °C) overnight.

Aliquots (≥250 µL) were then taken and transferred into 15 mL centrifuge tubes (Corning, Corning, NY, USA), which were centrifuged at 3000 rcf for 15 min at room temperature (Centrifuge 5702 RH, Eppendorf, Hamburg, Germany). Aliquots in chloroform were passed through a 0.2 µm filter (Acrodisc, Cytiva, Chicago, IL, USA) to remove particulates. Supernatant (50 µL) was taken to determine the amount of API that was in the supernatant. For MTX, this was done by HPLC after dilution with the mobile phase (as described below) and filtration through a 0.2 µm filter (Acrodisc). For insulin and lyophilized TT, the supernatant was dried in a vacuum oven at room temperature under a vacuum of −95 kPa for approximately 3 h prior to reconstitution in 0.1% *v*/*v* trifluoroacetic acid (TFA) (Sigma-Aldrich) in HPLC grade water (Sigma-Aldrich) (TFA–water) for insulin or with ~0.5% *v*/*v* Tween 20 (Sigma-Aldrich) in phosphate-buffered saline (MilliporeSigma, Burlington, MA, USA) (PBST) for lyophilized TT. Micro BCA assay (Thermo Fisher, Waltham, MA, USA) was then performed according to the manufacturer’s manual to determine the amount of insulin or TT that was in the supernatant. The working range was 50–800 µg/mL for TT, 133–1333 µg/mL for MTX, and 10–160 µg/mL for insulin. Additional dilutions were performed when results were higher than the calibration upper limit. While these tests did not determine the true solubilities of each API, they allowed us to identify solvents with poor API solubility. With these tests, amount of API that was dissolved (and/or possibly suspended as nano-sized particles since aliquots were filtered through a 0.2 µm filter) was determined for each API in each organic solvent.

Because suspensions can settle over time, we characterized the settling time of lyophilized TT suspended in 2-propanol with dissolved PEOX (Figure S2 in Supplementary Information), MTX suspended in acetone with dissolved PAA (Figure S3 in Supplementary Information), and insulin suspended in ACN with dissolved PVP (Figure S4 in Supplementary Information).

### 2.4. API Potency Retention After Exposure to Organic Solvent

To ensure that the API did not lose potency after exposure to organic solvents, samples of API (MTX and insulin) suspended in each organic solvent of interest were prepared at a 1 mg/mL concentration. For lyophilized TT, a lyophilized cake containing ~25 Lf TT and 5 mg sucrose was manually mixed in 400 µL of organic solvent of interest. Twenty microliters of the suspended lyophilized TT were cast on polydimethylsiloxane (PDMS) (Sylgard 184, Dow, Midland, MI, USA) chips. These suspensions in vials (for MTX and insulin) or on PDMS chips (for lyophilized TT) were then dried in a vacuum oven at room temperature under a vacuum of −95 kPa for 3 h. Lyophilized TT was reconstituted in PBST. MTX was reconstituted in 85% v/v 0.1% TFA–water and 15% *v*/*v* ACN. Insulin was reconstituted in 70% *v*/*v* 0.1% TFA–water and 30% *v*/*v* ACN. All MTX samples and insulin samples were then passed through a 0.2 µm filter. HPLC was performed to determine the amount of MTX and insulin recovered from each aliquot. An ELISA was performed to determine the TT potency remaining.

### 2.5. ELISA Analysis of TT

We performed competitive ELISA to determine TT potency based on a previously established protocol [25,26]. The ELISA analysis should be sensitive to structural and conformational changes at the antigenic site to which the anti-TT antibody binds. Stock TT solution (3480 Lg/mL) was diluted in carbonate buffer (1 capsule of carbonate–bicarbonate buffer (Sigma-Aldrich) in 100 mL DI water, pH 9.5) at a 1:500 ratio, and the diluted TT was coated onto a 96-well plate (Nunc-Immuno, Thermo Scientific, Waltham, MA, USA). The plate was sealed and refrigerated (2–8 °C) overnight. Samples and standards were prepared within the concentration range of 10.9–696 mLf/mL by dilution in PBST. Anti-TT horse radish peroxidase (HRP) antibody (Ab) (Alpha Diagnostic International, San Antonio, TX, USA) was also diluted at a 1:300 ratio in PBST and then mixed with each sample and standard at a 1:1 ratio. These mixtures were also refrigerated overnight.

After the overnight refrigeration, the plate was washed three times with PBST and then blocked with 30 mg/mL bovine serum albumin (Fisher Scientific, Pittsburgh, PA, USA) in carbonate buffer, sealed, and incubated for 1 h at 37 °C. The plate was then washed three times with PBST. The samples and standards mixture with anti-TT HRP Ab were then added to the plate. The plate was sealed and refrigerated overnight. The following day, the plate was washed six times with PBST, after which 100 µL KPL SureBlue 3,3′-5,5′-tetramethylbenzidine (TMB) Microwell Substrate (Seracare, Milford, MA, USA) was added to each well. The plate was sealed, covered with aluminum foil, and incubated at 37 °C for 15 min, after which 100 µL KPL TMB BlueSTOP (Seracare) was added to the plate. The plate was then read at 620 nm using an absorbance spectrophotometer (Biotek, Winooski, VT, USA). Overall, the assay took ~40 h, mostly at refrigerated temperature. Calibration curves were generated using linear fits of absorbance values from TT standards.

### 2.6. HPLC Analysis of MTX and Insulin

An HPLC system (1260 Infinity II LC System, Agilent Technologies) equipped with a quaternary pump, autosampler, and diode array detector was employed to quantify MTX and insulin concentrations. Chromatographic separation was achieved using a C18 column (ZORBAX Eclipse XDB-C18; 3.5 μm particle size, 4.6 mm internal diameter × 150 mm length, Agilent Technologies). The detailed operating parameters used for quantification are presented in Table S1 in the Supplementary Information. These HPLC analyses were designed primarily to determine API mass loading, recovery efficiency, and gross chemical stability, and may not be sensitive to molecular conformational changes, especially of insulin. All mobile phase solvents were of HPLC grade (Sigma-Aldrich).

Two HPLC methods were developed for the analysis of insulin. The first method was utilized to evaluate the amount of insulin in organic solvents, the recovery rate of insulin after exposure to organic solvents, and the compatibility of polymers with insulin, while the second method was optimized for the quantification of insulin in MNPs. The latter method provided improved separation of the insulin peak from interfering components originating from the skin.

Calibration curves and representative HPLC chromatograms are presented in Figures S5–S8 in Supplementary Information.

### 2.7. Polymer/Excipient Solubility in Organic Solvents

A selection of polymers was tested for their solubilities in organic solvents: cellulose acetate (CA), methyl cellulose (MC), carboxymethyl cellulose (CMC), hydroxyethyl cellulose (HEC), hydroxypropyl cellulose (HPC), (hydroxypropyl)methyl cellulose (HPMC), dextran (Dex), diethylaminoethyl-dextran hydrochloride (DEAE-Dex), polyacrylic acid (PAA), polyacrylamide (PAM), polyethylene oxide (PEO), poly(2-ethyl-2-oxazoline) (PEOX), polymethyl vinyl ether-alt-maleic acid (PMVE-MA), and PVP. All polymers were obtained from Sigma-Aldrich, other than Dex, which was obtained from ThermoFisher.

A selection of excipients was also tested for their solubilities in organic solvents: mannitol (VWR Chemicals, Solon, OH, USA), sucrose, glucose, dextrose (Spectrum Chemical, New Brunswick, NJ, USA), sorbitol (VWR Chemicals), mannose, maltose (Tokyo Chemical Industry, Portland, OR, USA), fructose, trehalose (Tokyo Chemical Industry), lactose (Spectrum Chemical), raffinose (Fluka Biochemika, Buchs, Switzerland), glycine, L-histidine (ThermoFisher), L-lysine, gelatin (from porcine skin), and hydroxypropyl-β-cyclodextrin (HPBC) (Tokyo Chemical Industry). All excipients were obtained from Sigma-Aldrich unless otherwise stated.

Excess amount of polymer or excipient was added to 2 mL of organic solvent in glass vials (Clear Borosilicate Vial). These were left on a shaker (Titramax 1000) at 300 rpm for one week at room temperature. At three timepoints during the week, 220 µL aliquots were taken if there was no floatation of samples or 250 µL aliquots were taken if there was floatation of samples (i.e., floatation was seen for all polymers and excipients in chloroform; CA in dioxane and water; MC in methanol and dioxane; HEC in ethanol; HPMC in methanol; dextran in toluene, dioxane, ethyl acetate, chloroform, ACN, and 2-propanol; PVP in toluene and ethyl acetate; PAM in toluene, dioxane, and ethyl acetate; PAA in chloroform). The 220 µL samples were added to a microcentrifuge tube (Eppendorf), while the 250 µL samples were added to a microcentrifuge tube with a filter (Ultrafree-MC Centrifugal Filter Units with a pore size of 5.0 µm, MilliporeSigma). Both the 220 µL and the 250 µL samples were centrifuged at 8160 rcf for 2 min (Eppendorf Centrifuge 5415 C). Then 200 µL of the supernatant was transferred to a microcentrifuge tube (Costar, Arlington, VA, USA) or a glass vial (Agilent, Santa Clara, CA, USA) and dried in a vacuum oven at room temperature under a vacuum of −95 kPa.

The difference in weights before supernatant was added and after supernatant was dried was then calculated to determine the amount of polymer or excipient that had been dissolved in the supernatant. Solubility was determined by the difference in weights once the weight stabilized or at the end of the one-week study, divided by the volume of the supernatant taken (200 µL). Solubility was then categorized as practically insoluble (<0.01% *w*/*v*), very slightly soluble (0.01–0.1% *w*/*v*), slightly soluble (0.1–1%), sparingly soluble (1–3.3% *w*/*v*), soluble (3.3–10% *w*/*v*), freely soluble (10–100% *w*/*v*), and very soluble (>100%) [27,28]. Additionally, all samples that were found to be insignificantly greater than 0 through a one-way *t*-test (*p* > 0.05, RStudio, version 1.4.1103, Boston, MA, USA) were categorized as practically insoluble.

### 2.8. Fabrication of MNP Molds

MNP molds were fabricated by casting PDMS (Sylgard 184) onto master structures to generate inverse molds in the shape of a microneedle array. MNP molds contained an array of 100 microneedles in an area of 1 cm^2^. Each microneedle was approximately 600 µm in height with a base diameter of approximately 300 µm and tip-to-tip distance of 1 mm.

### 2.9. Fabrication of MNPs Containing TT

Two lyophilized cakes (each containing ~50 Lf TT and 5 mg sucrose) were homogenized for 8 s in 800 µL of 2-propanol containing 9.5% *w*/*v* dissolved PEOX to make the casting solution. A volume of 50 uL was cast on each MNP mold that was placed on a vacuum chuck pulling a vacuum of −95 kPa at room temperature. The molds were then centrifuged at 2934 rcf for 90 min at 40 °C and then stored in a vacuum oven overnight at room temperature and −95 kPa. The next day, the molds were again placed on the vacuum chuck at room temperature, and ~200 µL of epoxy (Sevgili, Dongwan, China) was cast onto each mold and allowed to cure on the vacuum chuck overnight. After overnight curing, the molds were transferred to a vacuum oven and further dried at room temperature for 3 h at −95 kPa before demolding. The demolded MNPs were then stored in sealed aluminum pouches with desiccant (Drierite, Thermo Scientific) and stored under refrigeration until further use.

### 2.10. Fabrication of MNPs Containing MTX

A casting solution was prepared by suspending 1 mg/mL MTX in acetone containing 9% *w*/*v* dissolved PAA as a casting solution and vortexed before casting. MNP molds were placed on a vacuum chuck pulling a vacuum of −95 kPa at room temperature, and a volume of 20 µL of casting solution was applied to each mold every 30 min for a total of 60 µL. Thirty minutes after the last cast, ~200 µL of epoxy (Sevgili) was cast onto each mold, and the mold was left on the vacuum chuck overnight. The MNPs were demolded the following day and stored in sealed aluminum pouches with desiccant (Drierite) under refrigeration until further use.

### 2.11. Fabrication of MNPs Containing Insulin

A casting solution was prepared by suspending 1 mg/mL insulin in ACN containing 9.5% *w*/*v* dissolved PVP. A single 60 µL cast was applied onto each MNP mold that was on a vacuum chuck pulling a vacuum of −95 kPa at room temperature. After 2 h, ~136 mg UV glue (RapidFix, San Diego, CA, USA) was applied and spread across each MNP mold. UV light (RapidFix) was applied for 15 s to cure the UV glue.

### 2.12. MNP Characterization

To determine the insertion and delivery efficiency of MNPs, porcine ear skin (Pel-Freeze Biologicals, Rogers, AR, USA) was prepared ex vivo by shaving off the hair with a razor (Dyanarex, Orangeburg, NY, USA) and cleaning the skin with alcohol swabs (Becton, Dickinson and Company, Franklin Lakes, NJ, USA). The skin was then wiped dry before MNP insertion. During insertion, the MNP was firmly pressed into the skin by the thumb for 10 s, gently held in place by the thumb for 30 s, and then left in the skin for 15 min before removal. After removal, gentian violet solution (Humco, Texarkana, TX, USA) was applied at the site of administration to stain the sites of puncture into the skin. After 15 min, the gentian violet was wiped clean with alcohol swabs and tissues (Kimwipe). In this way, gentian violet staining provided a direct assessment of microneedle insertion into skin.

### 2.13. Statistical Analysis

GraphPad Prism software, version 10.3.1 (San Diego, CA, USA), was used for statistical analyses in this study, specifically to determine the mean and 95% confidence interval (CI) of samples and perform ANOVA analysis. For one-way ANOVA analysis, significance was defined as *p* < 0.05. RStudio version 1.4.1103 (Boston, MA, USA) was used for one-sided *t*-test analysis to determine solubility significantly greater than 0 (for polymer and excipient solubility).

## 3. Results

**Figure 1:**
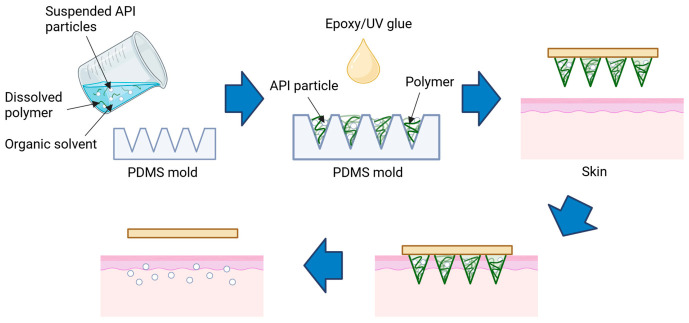
Schematic diagram of the process of MNP fabrication by API suspension casting and API delivery to the skin. A casting solution containing suspended API particles and dissolved polymer in an organic solvent is cast into a MNP mold. After drying to form microneedles comprised of a polymer matrix encapsulating API particles, epoxy or UV glue is applied to the mold to form the patch backing upon curing. The resulting microneedle patch is then pressed into the skin, where the polymer matrix dissolves and releases the API particles. Created in BioRender. Lu, C. (2026) https://BioRender.com/6casnlu, accessed on 14 April 2026.

Our goal was to develop a workflow for the rational design of MNP formulation and fabrication methods using suspension casting that incorporates API as a dry powder into the microneedles. Our strategy involves suspending API particles in an organic solvent in which the API does not dissolve, thereby keeping the API as a dry powder (Figure 1). We also select a polymer that is soluble in the organic solvent and in water. In this way, the polymer can form the microneedle matrix upon casting and drying in an MNP mold and then can dissolve away to release the API upon insertion into the skin.

**Figure 2:**
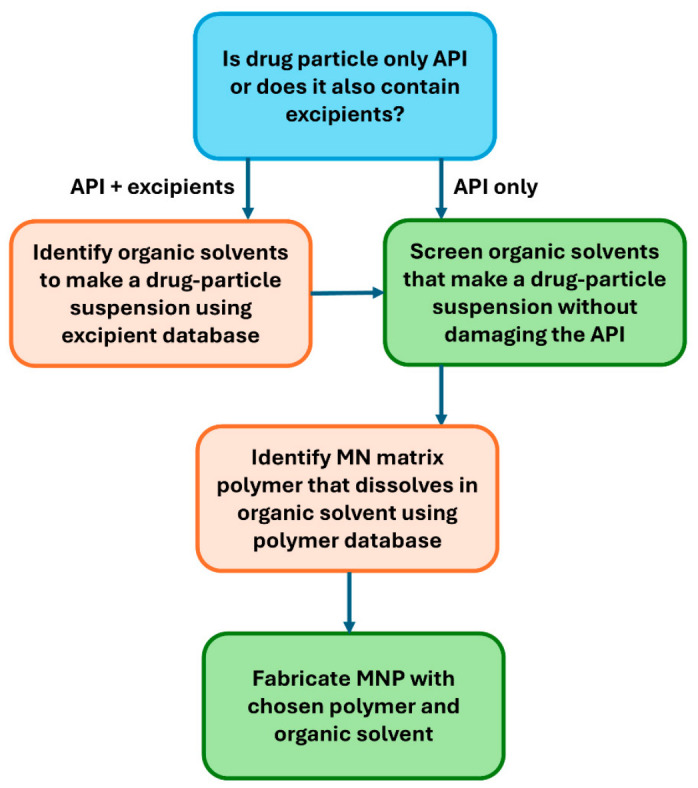
Flowchart describing the process for rational design of dissolving MNPs fabricated by suspension casting of dry powder APIs.

Design of this fabrication process requires selection of suitable organic solvents for casting, polymers to form the microneedle matrix, and other excipients to stabilize the API. As shown in Figure 2, the first step is to determine if the API particles contain excipients. Either way, we need to select a solvent that will not dissolve the API and also not dissolve any excipients that are present. If excipients are needed to stabilize the API particles, then the second step is to select suitable excipients using a database we developed that identifies excipients that do not appreciably dissolve in candidate solvents.

The third step is to screen organic solvents that do not dissolve or otherwise damage the API particles during suspension and subsequent drying. As a fourth step, we developed a database of polymers that are both water-soluble and soluble in various organic solvents, which identifies polymers that can be used with the organic solvent selected for API suspension. Finally, we use the API/excipient/polymer/organic solvent system as a casting solution to fabricate microneedles and verify their functionality to retain API potency and deliver API into the skin.

### 3.1. API Compatibility with Organic Solvents

In this study, we used the proposed workflow to develop dissolving MNPs for administration of a model vaccine (TT), small-molecule drug (MTX) and biologic drug (insulin). We first selected nine candidate organic solvents from among those commonly used in the chemical and pharmaceutical industries [29]. These solvents were down-selected to encompass a range of different relative polarities and to have boiling temperatures close to or lower than that of water (i.e., 100 °C). We wanted to include a broad range of polarities because the ability of the organic solvent not to dissolve the API particles, possibly including excipients, while also being able to dissolve the water-soluble polymer needed for the microneedle matrix, may depend on achieving the right balance of solvent polarity. We wanted a solvent with a boiling point that is sufficiently low to expedite drying during MNP fabrication, but not so low that the solvent evaporates during handling. We found that a boiling point near that of water achieves this balance well. The final selection of solvents was (from least polar to most polar): toluene, dioxane, ethyl acetate, chloroform, acetone, ACN, 2-propanol, ethanol, and methanol (see Table S2 in Supplementary Information for solvent polarity, boiling temperature, and density).

### 3.2. Lyophilized TT Suspension

**Figure 3:**
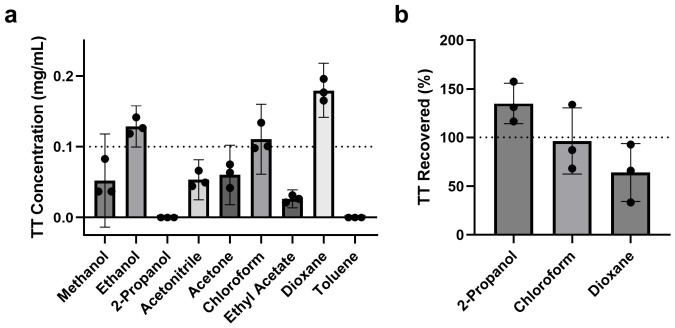
Tetanus toxoid (TT) concentration and stability after suspension in organic solvents. (**a**) TT concentration in the supernatant of organic solvent after suspension and overnight shaking at room temperature determined by micro BCA assay. The dashed line indicates a concentration of 0.1 mg/mL, which is defined as practically insoluble. Solvents for which the upper limit of the 95% confidence interval of TT concentration falls below 100 µg/mL were classified as practically insoluble. (**b**) Percentage of antigenic TT recovered after exposure to organic solvents, as determined by ELISA. Dashed line is for 100% recovery. Results are reported as mean ± 95% confidence interval (*n* = 3) (Ordinary one-way ANOVA for Figure 3b: *p* > 0.05).

The first API that we studied was TT. TT is a vaccine that prevents tetanus, which is a potentially fatal disease caused by toxins produced by *Clostridium tetani* [29]. In this study, TT was lyophilized with sucrose (Figure S1a in Supplementary Information), and the lyophilized TT was suspended in the nine candidate organic solvents. The following day, TT dissolution was measured in each organic solvent to identify which ones suspended the TT particles without dissolving them (Figure 3a). Based on the United States Pharmacopeia definition for practically insoluble (<0.1 mg/mL) [27], we identified 2-propanol, ACN, ethyl acetate, and toluene as non-solvents for TT, with 2-propanol and toluene having no detectable solubilization. Dioxane and ethanol had the highest TT concentration, although they were still relatively low (i.e., <0.2 mg/mL).

To test our hypothesis that TT stability correlated with its inability to dissolve in a solvent, we measured the antigenic TT recovery rate (as determined by ELISA) in three of the organic solvents: one with no detectable dissolution (2-propanol), one with TT concentration near the threshold for practically insoluble (chloroform), and one well above that threshold (dioxane) (Figure 3b). While there was a negative trend of TT potency after suspension with increasing TT concentration in the organic solvent, the differences were not statistically significant (*p* > 0.05, one-way ANOVA). These data suggested that 2-propanol would be the best choice for making suspension-cast MNPs with TT.

### 3.3. MTX Suspension

**Figure 4:**
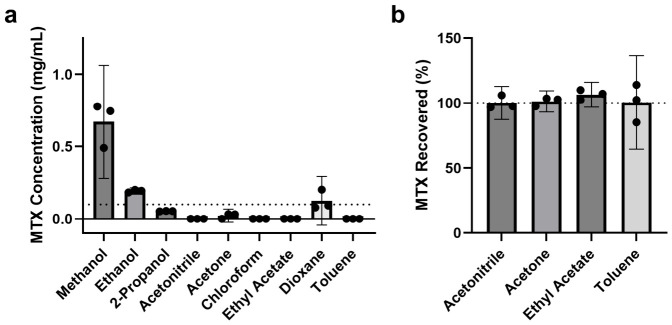
MTX concentration and stability after suspension in organic solvents. (**a**) MTX concentration in the supernatant of organic solvent after suspension and overnight shaking at room temperature. The dashed line indicates a concentration of 100 µg/mL, which is defined as practically insoluble. Solvents for which the upper limit of the 95% confidence interval of MTX concentration fell below 0.1 mg/mL were classified as practically insoluble. (**b**) Percentage of MTX recovered after exposure to organic solvents. Dashed line is for 100% recovery rate. MTX concentration was determined by HPLC. Results are reported as mean ± 95% confidence interval (*n* = 3).

We next considered organic solvents suitable for MTX formulation. MTX is a small-molecule drug that can be used to treat a variety of indications, including cancer and autoimmune inflammatory diseases such as rheumatoid arthritis [30]. Multiple published works have addressed fabricating dissolving MNPs for MTX delivery using other MNP designs [31,32,33]. This is because MTX is used to treat skin conditions like psoriasis, making local delivery to the skin favored over systemic delivery, which can have toxic side effects, especially in the gastrointestinal tract [34]. MTX concentration in almost all of the organic solvents was below the practically insoluble limit and was only higher in methanol, ethanol, and dioxane (Figure 4a). There was essentially no measurable amount of MTX in ACN, acetone, chloroform, ethyl acetate, and toluene. After suspending MTX in four of these solvents, MTX retained full potency after drying (Figure 4b) and appeared to retain similar particle size and morphology (suspension in acetone, Figure S9b,c in Supplementary Information), indicating that any of these solvents would be suitable for MNP fabrication with MTX.

### 3.4. Insulin Suspension

**Figure 5:**
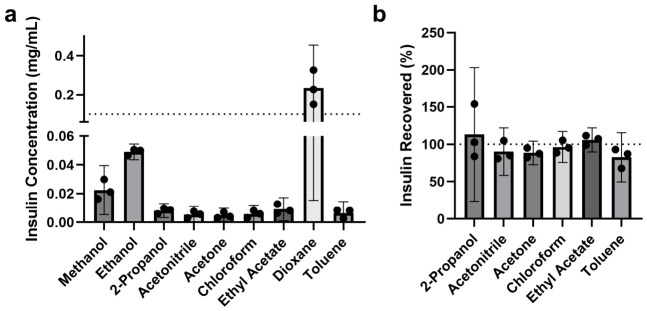
Insulin concentration and stability after suspension in organic solvents. (**a**) Insulin concentration in the supernatant of organic solvent after suspension and overnight shaking at room temperature. The dashed line indicates a concentration of 100 µg/mL, which is defined as practically insoluble. Solvents for which the upper limit of the 95% confidence interval of insulin concentration fell below 0.1 mg/mL were classified as practically insoluble. Insulin concentration was measured through micro BCA assay. (**b**) Percentage of insulin recovered after exposure to organic solvent. Dashed line is for 100% recovery rate. Insulin concentration was determined by HPLC. Results are reported as mean ± 95% confidence interval (*n* = 3).

As a representative biologic drug, we assessed solvents for insulin, which is widely used to treat diabetes [35]. We found that insulin concentration was below the practically insoluble limit in all of the organic solvents except dioxane (Figure 5a). Insulin concentration was higher in ethanol and methanol compared to other organic solvents, but all were well below the practically insoluble limit. Insulin had no significant loss of potency after suspension and drying in 2-propanol, ACN, acetone, chloroform, ethyl acetate, and toluene (Figure 5b) and appeared to retain similar particle size and morphology (suspension in ACN, Figure S9d,e in Supplementary Information), providing multiple organic solvents suitable for MNP fabrication.

### 3.5. Excipient Compatibility with Organic Solvents

Among the above three APIs evaluated, TT was stable when formulated with sucrose, while MTX and insulin were stable as received from the manufacturer without added excipients. However, other APIs might need to be stabilized through formulation, so we screened 16 excipients that are commonly used for drug stabilization during lyophilization (e.g., sugars, amino acids) [36,37] to determine their solubilities in our candidate organic solvents. We categorized each excipient–solvent combination according to its solubility, ranging from practically insoluble to very soluble, as shown in Table 1.

Most excipients in our database did not dissolve well in the organic solvents, ranging from slightly soluble to practically insoluble (Table 2). Among all of the organic solvents, methanol (i.e., the most polar solvent) was the best solubilizer of the excipients, while toluene (i.e., the least polar solvent) was the worst. Among all of the excipients, HPBC had the best overall solubility in the organic solvents, while glycine and lactose had the worst overall solubility.

### 3.6. Polymer Compatibility with Organic Solvents

After identifying solvents that were compatible with APIs and their formulation excipients, we next needed to identify polymers that are soluble in the candidate solvents, can serve as the matrix material for mechanically strong microneedles, and can dissolve in the aqueous environment of the skin. We selected a database of 14 polymers commonly used as the matrix material for dissolving microneedles, such as CMC [38], dextran [39], PVP [40], PAA [41], and PMVE-MA [42], as well as their derivatives, and other polymers with a similar structure, and determined their solubility in our selection of organic solvents (Table 3). Among all of the polymers tested, PEOX was soluble in the largest number of organic solvents, followed by HPC, PVP and PAA, indicating that these polymers should be good choices to form the microneedle matrix during suspension casting. In general, the cellulose derivatives were less soluble in the organic solvents compared to the other polymers. Among the organic solvents, the more polar ones (i.e., alcohols) were generally better at dissolving the synthetic polymers (i.e., not cellulose derivatives) than the less polar solvents. None of the solvents was especially good at dissolving the cellulose derivatives. Toluene was a uniformly bad solvent for all polymers tested.

### 3.7. Fabrication of MNPs by Suspension Casting in Organic Solvent

Our screening produced solvent + excipient combinations suitable for each of the three APIs. Our next step was to make MNPs using these formulations.

### 3.8. Lyophilized TT MNPs

**Figure 6:**
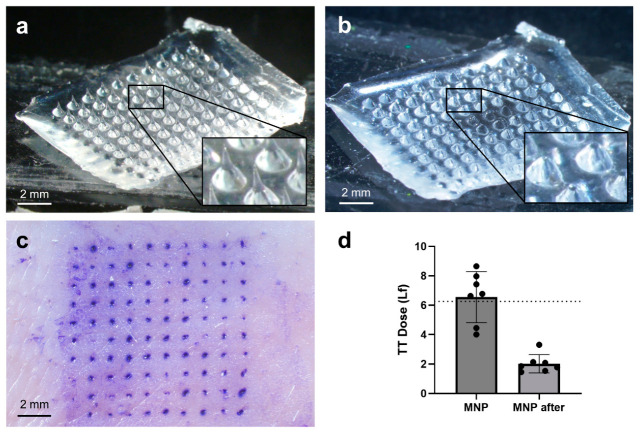
TT microneedle patches. Representative microscopic images of a TT MNP (**a**) before and (**b**) after insertion into ex vivo porcine skin, and (**c**) the administration site of the same MNP on the skin after staining sites of microneedle puncture with gentian violet dye. Insets are 3× magnified. (**d**) TT dose encapsulated in MNPs before and after insertion into ex vivo porcine skin. MNPs were fabricated with a casting solution containing 9.5% *w*/*v* PEOX in 2-propanol. Results are reported as mean ± standard deviation (*n* = 7). The expected dose (6.3 Lf) per MNP is indicated by the dashed line.

To make TT MNPs, we selected 2-propanol as the solvent and PEOX as the polymer to make the microneedle matrix. PEOX was selected because it is soluble in many of the organic solvents tested and, therefore, may be a broadly applicable polymer for MNP fabrication using organic solvent casting, and 2-propanol was selected as a solvent that appeared not to damage TT or dissolve the sucrose in which TT was formulated during prior lyophilization. Lyophilized TT was suspended in 2-propanol with dissolved PEOX and cast onto MNP molds (Figure 6a). Upon application to the skin, the microneedles dissolved after insertion (Figure 6b) and were able to puncture through the stratum corneum (Figure 6c). The MNPs were fabricated with an average dose of 6.6 ± 1.7 Lf per patch and delivered 4.6 Lf into the skin (Figure 6d), which is close to the dose of TT found in many approved human vaccines (i.e., 5 Lf of TT) [43]. The microneedle insertion efficiency was determined to be 97 ± 2% (*n* = 5) (Figure 6c), and the TT delivery efficiency was 69 ± 9% (*n* = 7) (Figure 6d), based on an expected dose of 6.3 Lf.

### 3.9. MTX MNPs

**Figure 7:**
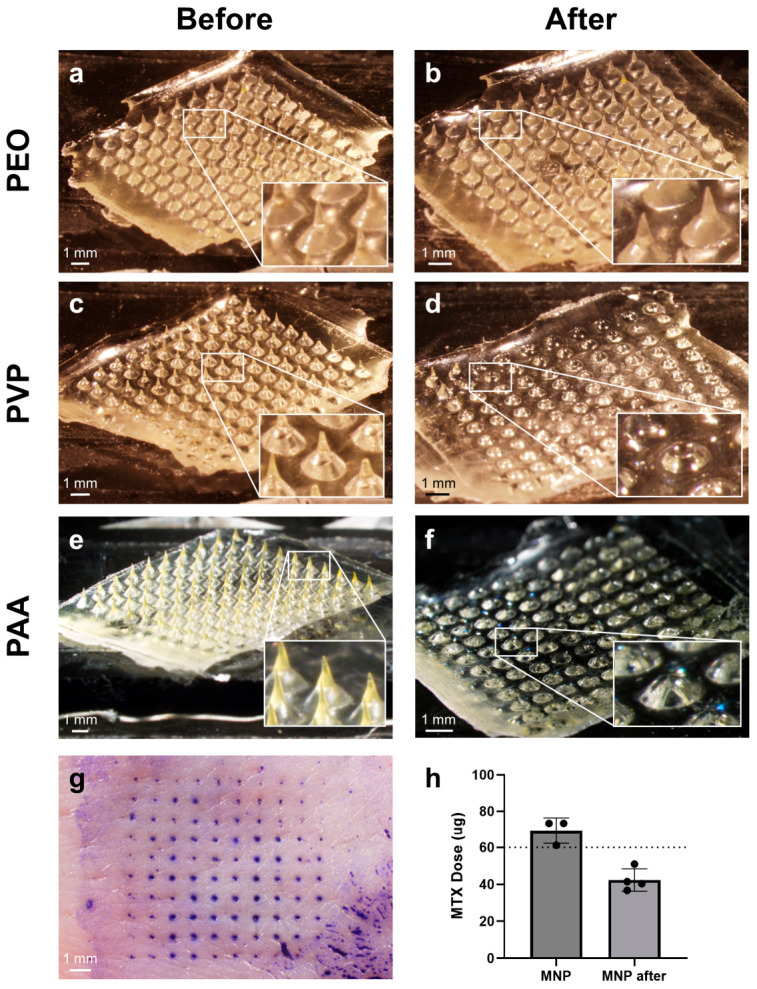
MTX microneedle patches. Representative microscopic images of a MTX MNP fabricated with a casting solution of 8% *w*/*v* PEO in ACN (**a**) before and (**b**) after reconstitution in 75% TFA–water and 25% ACN; a MTX MNP fabricated with a casting solution of 9% *w*/*v* PVP in ACN (**c**) before and (**d**) after reconstitution in 75% TFA–water and 25% ACN; a MTX MNP fabricated with a casting solution of 9% *w*/*v* PAA in acetone (**e**) before and (**f**) after insertion into ex vivo porcine skin; and (**g**) the skin insertion site of a PAA-based MNP stained with gentian violet dye to identify sites of skin puncture. Insets are 3× magnified. (**h**) MTX dose encapsulated in PAA-based MNPs before and after insertion into ex vivo porcine skin. Results are reported as mean ± standard deviation (n ≥ 3). The expected dose per MNP was 60 µg, as indicated by the dashed line.

To make MTX MNPs, we used results from the MTX suspension experiments and the polymer solubility database to identify formulations of PAA in acetone, PEO in ACN, and PVP in ACN as good candidates for fabricating MTX MNPs. We selected these candidates to demonstrate that additional polymers (i.e., other than PEOX) and organic solvents (e.g., other than 2-propanol) can also be used for MNP fabrication using organic solvent casting. In an initial screening, all three formulations had no loss of MTX potency after casting and drying on PDMS chips (Figure S10 in Supplementary Information). However, when making MNPs, the microneedles made using PEO in ACN did not dissolve after reconstitution (Figure 7a,b). This suggests that the epoxy backing may have flowed into the microneedle portion of the MNP mold during manufacturing and made the microneedles non-dissolving. After casting with the PVP-in-ACN formulation, there were many broken microneedles, although the microneedles were able to dissolve (Figure 7c,d). The presence of broken microneedles suggests that the microneedles may have been too brittle for demolding at the end of manufacturing. Only MNPs made with PAA in acetone had no broken microneedles and could insert into skin and dissolve after insertion (Figure 7e–g). Therefore, MTX MNPs were fabricated using PAA in acetone with a dose of 69 ± 7 µg (Figure 7h) and had an insertion efficiency of 97 ± 2% (*n* = 5) (Figure 7g) and a delivery efficiency of 39 ± 9% (*n* = 4) (Figure 7h), based on an expected dose of 60 µg.

### 3.10. Insulin MNPs

**Figure 8:**
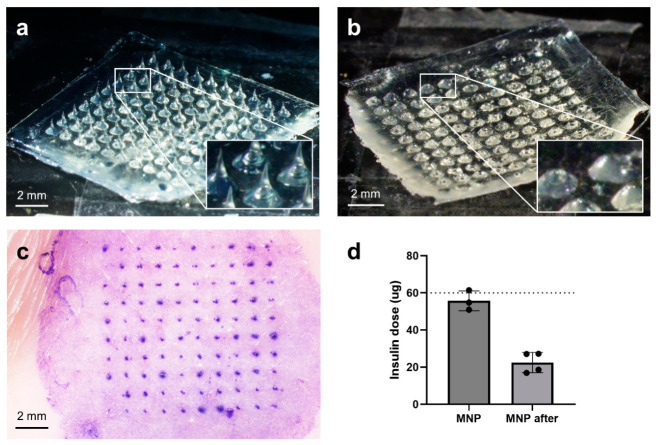
Insulin microneedle patches. Representative microscopic images of an insulin MNP (**a**) before and (**b**) after ex vivo porcine skin insertion, and (**c**) the administration site of the same insulin MNP on the skin after staining sites of microneedle puncture with gentian violet dye. Insets are 3× magnified. (**d**) Insulin dose encapsulated in insulin MNPs before and after insertion into ex vivo porcine skin. MNPs were fabricated with a casting solution containing 9.5% *w*/*v* PVP in ACN. Results are reported as mean ± standard deviation (n ≥ 3). The expected dose per MNP was 60 µg, as indicated by the dashed line.

To make insulin MNPs, we selected two possible formulations for fabrication: PEO in ACN and PVP in ACN. We selected these combinations because they provided new polymer/solvent systems that were not used to make the TT or MTX MNPs. Both formulations produced no significant loss of insulin potency after casting and drying on PDMS chips in a screening study (Figure S11 in Supplementary Information). However, after fabricating insulin MNPs using PEO in ACN, the microneedles did not dissolve completely after reconstitution (Figure S12 in Supplementary Information). Therefore, the PVP-in-ACN formulation was used to make insulin MNPs (Figure 8a) with a dose of 56 ± 5 µg (*n* = 3) (Figure 8d). These MNPs were able to insert into the skin (Figure 8c) and dissolve after insertion (Figure 8b). After insertion, the dose remaining on the MNP was 23 ± 5 µg (*n* = 4) (Figure 8d). Overall, the insulin MNPs had an insertion efficiency of 93 ± 3% (*n* = 3) and an insulin delivery efficiency of 60 ± 10% (*n* = 4), based on an expected dose of 60 µg.

## 4. Discussion

In this study, we established a new approach for fabricating dissolving MNPs through suspension casting. The benefit of this approach is that it avoids the dissolution and drying of APIs that takes place during typical dissolving MNP fabrication. This protects the API from damage during drying and avoids the need to formulate microneedle casting solutions with excipients added to protect the API during drying. Additionally, our approach enables rational design of the polymer and organic solvent used in MNP fabrication based on concentration measurements in various organic solvents in the databases we created. We demonstrated the utility of our approach by designing and fabricating dissolving MNPs that encapsulated a lyophilized vaccine (i.e., TT), small-molecule drug (i.e., MTX), and biologic drug (i.e., insulin). While specific formulation results from this study can guide future formulations, we believe that this study developed a streamlined method for identification of possible solvent/polymer/excipient combinations to fabricate MNPs for APIs broadly.

At its core, this was a study of solubility. We needed to match polymers, excipients and APIs with organic solvents that enabled (i) the polymers to dissolve in both the organic solvent and in the aqueous skin, (ii) the excipients not to dissolve in the organic solvent, but to dissolve in the skin, and (iii) the API not only not to dissolve but not even interact with the organic solvent in a way that could damage the API. While we did not determine their true solubilities, we measured the concentration of polymers, excipients, and APIs that were dissolved or possibly nano-suspended in each organic solvent. In this way, we were able to find polymer/excipient/API/solvent combinations that produced MNPs that encapsulated the active drug and delivered it into the skin.

### 4.1. Polymer Solubility Database

One of the challenges of this study was to identify polymers that were hydrophobic enough to dissolve in an organic solvent during MNP fabrication and hydrophilic enough to dissolve in the aqueous fluids of the skin. While polar solvents would be expected to be more suitable for dissolving water-soluble polymers, we could not necessarily use the most polar solvents because they also needed to not dissolve API and excipients, which would generally be less soluble in non-polar solvents.

To identify suitable solvents, we measured the organic-solvent solubility of 14 water-soluble polymers commonly used for dissolving MNP fabrication and their derivatives. This was done by determining the concentration of polymers that were dissolved and/or possibly suspended as nanoparticles in each organic solvent. Examination of our polymer database showed that toluene was a poor solvent for all of the tested polymers, indicating that solvents with such a low polarity index may be unsuitable for dissolving MNP fabrication. In general, the synthetic polymers exhibited greater solubility in a larger number of solvents compared to the cellulose-based polymers. This may be explained by the many hydroxyl groups on cellulose that lead to strong intramolecular and intermolecular hydrogen bonding that cannot be easily broken by organic solvents, making solvent penetration difficult [44,45]. Cellulose also has crystalline regions, which further inhibit its dissolution and is known to have poor solubility in water [46] and organic solvents [44,45]. In contrast, the synthetic polymers in this study should have weaker intramolecular or intermolecular forces, less crystallinity, and stronger interaction between polymer and liquid [46].

### 4.2. Excipient Solubility Database

The 16 excipients in our database were selected as ones that are commonly used in lyophilization, which resulted in mostly sugars and a few amino acids and macromolecules. Because lyophilized drug products are generally designed to quickly dissolve in water upon reconstitution, these excipients were generally water-soluble and polar, which resulted in uniformly low solubility in organic solvents. This was favorable for our objective of having excipients with high solubility in water and low solubility in organic solvents.

The main exceptions to this trend of low solubility were combinations that included methanol or HPBC. This may be explained by the fact that methanol was the most polar organic solvent, which would be expected to solubilize water-soluble excipients better than the other organic solvents. HPBC is known to solubilize poorly soluble drugs and cholesterol due to the presence of hydroxypropyl groups that disrupt the hydrogen bonding network of the hydroxyl groups comprising the cyclodextrin backbone [47]. Our data was also consistent with a published paper on sugar solubility in alcohols, which found that fructose has a higher alcohol solubility than glucose and lactose, and that methanol provides higher solubility than ethanol or 2-propanol for these sugars [48].

While excipient–solvent combinations would ideally be practically insoluble, MNP fabrication with lyophilized TT formulated with sucrose and cast using 2-propanol was successful, despite sucrose being very slightly soluble in 2-propanol. This suggests that excipient–solvent combinations that are very slightly soluble and practically insoluble may be suitable for making suspensions for MNP fabrication.

### 4.3. TT MNPs

In TT suspensions, we found that the percentage of antigenic TT recovered negatively correlated with the total amount of TT found in the organic solvent, which suggests that TT that dissolved and/or nano-suspended in organic solvent may have lost activity. In this study, we fabricated TT MNPs using a suspension of lyophilized TT in 2-propanol with dissolved PEOX. There has been limited use of PEOX as the microneedle matrix material despite its biocompatibility and tuneability [49]. One study found that blends of PEOX were required for MNP fabrication when using PEOX of low molecular weight [49]. Since we used PEOX of a higher molecular weight (500 kDa), we were able to fabricate microneedles without the need for a blend.

Prior studies have investigated fabricating dissolving MNPs for TT vaccine delivery [26,50,51,52] because MNPs can increase TT vaccination coverage in low-resource settings due to their simplified administration compared to conventional intramuscular injections [11]. These studies used conventional micromolding techniques with an aqueous cast containing TT [26,50,51,52], and did not investigate using an organic solvent cast containing suspended lyophilized TT, like in the current study. We avoided the use of any water in our casting formulations containing TT because moisture exposure has been shown to affect the antigenicity of lyophilized TT [53].

### 4.4. MTX MNPs

We also assessed suspension-casting MTX to form MNPs to contain a representative small-molecule drug. These MNPs were fabricated using MTX suspended in acetone with dissolved PAA. Multiple published works have investigated fabricating dissolving MNPs for MTX delivery [31,32,33]. All of these studies used an aqueous casting formulation to make their MTX MNPs [31,32,33]. One study also used PAA to form the microneedle matrix, but the fabrication process took 3–4 days of drying [31]. Our suspension-casting fabrication method, combined with epoxy backing, enabled fabrication of MTX MNPs within 24 h, which shows another possible benefit of using a volatile, nonaqueous cast.

### 4.5. Insulin MNPs

As a representative biologic drug, we fabricated insulin MNPs by suspending insulin in ACN with dissolved PVP. There are many published papers on dissolving MNPs for insulin delivery [1,2,54], motivated by the ease of use and lack of pain during the frequent insulin administration required for diabetic patients [11] in comparison to conventional subcutaneous injections for insulin delivery [55]. These prior studies all used an aqueous cast during the fabrication of MNPs [1,2,54], and most of them required at least a day of drying [1,2] as opposed to the ~2 h fabrication time for the insulin MNPs presented in this study.

### 4.6. Limitations and Future Work

While suspension-casting in organic solvents simplified MNP fabrication design by removing the need to stabilize APIs during the dissolution and drying process associated with aqueous solution casting, it also complicated MNP fabrication design by requiring the selection of solvents, polymers, and excipients that have suitable solubility properties. The rational design approach presented here, guided by the polymer and excipient solubility databases, was developed to facilitate the design process, but more work is needed to expand the solvents, polymers, and excipients in the databases and to offer predictive models for formulation design. We only investigated suspension-casting of three APIs and only characterized them in vitro. Future studies should expand the scope of APIs studied and progress their evaluation into animal studies of pharmacokinetics, pharmacodynamics and safety.

Removal of residual solvent in our suspension-casting manufacturing method is important for future use in humans. Future studies need to measure residual solvent and optimize manufacturing methods to keep it at safe levels. Although we did not directly measure residual solvent levels in the MNPs fabricated in this study, we made estimates of the maximum amount of residual solvent and determined that it was well below the safety limits found in the International Council of Harmonisation of Technical Requirements for Pharmaceuticals for Human Use (ICH) Q3C(R9) Guideline for Residual Solvents [56]. According to ICH guidelines, the maximum daily intakes for the solvents used in this study—2-propanol, acetone, and ACN—are 50 mg, 50 mg and 4.1 mg, respectively. To determine an upper limit on the amount of solvent that could be in the MNPs, we multiplied the combined volume of the 100 microneedles per MNP (i.e., 1.4 µL) by the density of each solvent (i.e., 0.785 g/mL, 0.785 g/mL, and 0.786 g/mL, respectively) to determine the maximum amount of solvent that could be cast into a MNP mold (i.e., 1.099 mg, 1.099 mg, and 1.100 mg, respectively). The actual solvent volume cast used during MNP manufacturing would be less, and the residual solvent would be much less, indicating that residual solvent in the MNPs in this study should be well within ICH safety guidelines.

## 5. Conclusions

In this study, we developed a novel approach to fabricating dissolving MNPs by suspending drug particles in organic solvents and studied it in the context of combinations of 3 APIs, 9 organic solvents, 14 biocompatible polymers and 16 stabilizing excipients. To facilitate development of formulations and fabrication methods to make MNPs using this approach, we first identified organic solvents that can suspend each API without dissolution. We then created databases to identify excipients (for possible co-formulation with APIs to stabilize them) that do not dissolve in selected organic solvents and polymers (to form a strong microneedle matrix) that do dissolve in the selected organic solvents. Guided by this solubility information, we were able to fabricate MNPs for lyophilized TT, MTX, and insulin with this suspension-casting approach.

## Figures and Tables

**Table 1:** Categories and color scheme for solubility database ^1^.

Practically Insoluble	Very Slightly Soluble	Slightly Soluble	Sparingly Soluble	Soluble	Freely Soluble	Very Soluble
<0.01%	0.01–0.10%	0.1–1.0%	1.0–3.3%	3.3–10%	10–100%	>100%

^1^ Percentage expressed is % *w*/*v*.

**Table 2:** Solubility of excipients in organic solvents ^1^.

	Toluene	Dioxane	Ethyl Acetate	Chloroform	Acetone	Acetonitrile	2-Propanol	Ethanol	Methanol
Mannitol	0.00 ± 0.00	0.08 ±0.10	0.15 ±0.05	0.13 ±0.08	0.03 ±0.01	0.00 ±0.09	0.00 ±0.00	0.08 ±0.12	0.10 ±0.05
Sucrose	0.02 ±0.03	0.02 ±0.10	0.22 ±0.03	0.23 ±0.08	0.03 ±0.02	0.05 ±0.09	0.07 ±0.03	0.12 ±0.08	0.57 ±0.10
Glucose	0.02 ±0.03	0.13 ±0.03	0.25 ±0.09	0.03 ±0.03	0.00 ± 0.01	0.03 ± 0.06	0.13 ±0.08	0.38 ±0.14	1.82 ±0.10
Dextrose	0.03 ±0.03	0.13 ±0.06	0.07 ±0.08	0.05 ±0.00	0.07 ±0.06	0.05 ±0.05	0.13 ±0.06	0.30 ±0.05	1.82 ±0.41
Sorbitol	0.02 ±0.03	0.37 ±0.06	0.20 ±0.09	0.07 ±0.08	0.13 ±0.06	0.18 ±0.03	0.50 ±0.05	0.87 ±0.03	2.62 ±0.18
Mannose	0.02 ±0.03	0.43 ±0.03	0.23 ±0.19	0.03 ±0.03	0.10 ±0.05	0.18 ±0.08	0.55 ±0.00	1.15 ±0.13	>4.30 ±0.80 *
Maltose	0.15 ±0.00	0.10 ±0.05	0.00 ±0.00	0.00 ±0.00	0.02 ±0.01	0.17 ±0.03	0.23 ±0.06	0.53 ±0.08	>7.28 ±0.47 *
Fructose	0.13 ±0.03	0.53 ±0.08	0.07 ±0.03	0.15 ±0.10	0.13 ±0.06	0.08 ±0.08	0.77 ±0.10	1.68 ±0.03	>10.97 ±1.32 *
Trehalose	0.02 ±0.03	0.07 ±0.03	0.03 ±0.03	0.03 ±0.03	0.10 ±0.05	0.10 ±0.05	0.11 ±0.03	0.45 ±0.00	0.27 ±0.06
Lactose	0.08 ±0.10	0.03 ±0.06	0.03 ±0.06	0.12 ±0.08	0.00 ± 0.03	0.00 ± 0.05	0.05 ±0.05	0.00 ± 0.10	0.10 ±0.05
Raffinose	0.05 ±0.09	0.07 ±0.03	0.02 ±0.03	0.08 ±0.08	0.00 ± 0.15	0.00 ±0.00	0.15 ±0.05	0.33 ±0.03	>8.32 ±0.58 *
Glycine	0.00 ±0.05	0.05 ±0.05	0.00 ±0.05	0.07 ±0.08	0.00 ± 0.05	0.08 ±0.06	0.08 ±0.03	0.00 ± 0.00	0.00 ±0.00
Histidine	0.00 ± 0.06	0.05 ±0.05	0.07 ±0.03	0.12 ±0.03	0.00 ± 0.03	0.02 ±0.03	0.08 ±0.03	0.00 ± 0.06	0.03 ±0.06
Lysine	0.05 ±0.05	0.02 ±0.10	0.07 ±0.03	0.18 ±0.08	0.00 ± 0.03	0.06 ±0.03	0.15 ±0.09	0.35 ±0.13	0.97 ± 0.08
Gelatin	0.00 ± 0.00	0.00 ± 0.03	0.08 ±0.03	0.15 ±0.00	0.02 ±0.06	0.20 ±0.05	0.05 ±0.05	0.05 ±0.00	0.07 ±0.06
HPBC	0.08 ±0.06	1.50 ±0.18	0.15 ±0.05	0.08 ± 0.08	0.05 ±0.05	0.00 ±0.15	2.30 ±0.30	>1 g/mL *	>1 g/mL *

^1^ Excipient concentration is expressed as mean ± standard deviation (*n* = 3) with units of % *w*/*v*. Concentrations represent excipient that was dissolved and/or possibly suspended as nano-sized particles. The definition and color scheme for solubility categories is provided in Table 1. * Sample appeared fully dissolved.

**Table 3:** Solubility of polymers in organic solvents ^1^.

	Toluene	Dioxane	Ethyl Acetate	Chloroform	Acetone	Acetonitrile	2-Propanol	Ethanol	Methanol	Water
CA	0.28 ± 0.21	>4.37 ±1.42 *	5.22 ±1.44	0.52 ±0.06	>10.43 ±2.88	1.97 ± 0.13	0.02 ±0.03	0.03 ±0.03	0.00 ±0.00	2.17 ± 0.60
MC	0.13 ±0.11	0.37 ±0.25	0.05 ±0.05	0.17 ±0.16	0.42 ±0.14	0.33 ±0.03	0.28 ±0.06	0.02 ±0.03	0.18 ±0.03	>1.72 ± 0.63 *
CMC	0.00 ±0.00	0.27 ±0.12	0.03 ±0.03	0.25 ±0.05	0.00 ±0.05	0.10 ±0.05	0.03 ±0.06	0.08 ±0.10	0.00 ±0.00	>8.60 ±3.93 *
HEC	0.10 ±0.05	0.13 ±0.18	0.32 ±0.08	0.25 ±0.00	0.40 ±0.07	0.03 ±0.09	0.18 ±0.10	0.55 ±0.18	1.62 ±0.20	>5.65 ±0.69 *
HPC	0.17 ±1.15	>7.60 ±1.78 *	0.47 ±0.13	>4.77 ±1.81 *	2.13 ±0.54	1.15 ±0.26	>5.43 ±1.81 *	>13.32 ±1.04 *	>13.03 ±3.75 *	>6.12 ±0.50 *
HPMC	0.48 ±0.33	0.07 ±0.03	0.03 ±0.08	0.35 ±0.05	0.58 ±0.19	0.13 ±0.19	0.07 ±0.03	0.23 ±0.15	0.70 ±0.13	>1.22 ±0.11 *
Dex	0.00 ±0.00	0.08 ±0.04	0.02 ±0.03	0.03 ±0.04	0.00 ± 0.06	0.05 ±0.05	0.10 ±0.09	0.00 ±0.06	0.07 ±0.05	>27.88 ±0.90 *
DEAE-dex	0.08 ±0.03	0.32 ±0.03	0.28 ±0.08	0.38 ±0.23	0.07 ±0.08	0.07 ±0.08	0.00 ±0.16	0.00 ±0.10	>13.27 ±6.24 *	>31.37 ±2.11 *
PAM	0.13 ±0.08	0.00 ±0.15	0.10 ±0.09	0.17 ±0.03	0.00 ±0.00	0.13 ±0.03	0.05 ±0.05	0.07 ±0.03	0.00 ±0.00	>21.60 ±1.65 *
PAA	0.00 ±0.00	>9.85 ±0.18 *	0.92 ±0.14	0.28 ±0.10	11.87 ±2.62	0.52 ±0.30	>16.72 ±1.77 *	>33.95 ±15.52 *	>16.45 ±10.23 *	>34.63 ±2.11 *
PEOX	0.03 ±0.06	>8.58 ±2.53 *	8.08 ±2.18 *	>35.07 ±7.06 *	>26.10 ±6.36 *	>23.50 ±3.46 *	>14.48 ±2.44 *	>15.50 ± 0.36 *	>17.43 ±2.44 *	>32.95 ±4.28 *
PEO	0.22 ±0.03	0.37 ±0.26	0.18 ±0.10	7.88 ±0.21	1.05 ±0.13	8.33 ±3.33	0.47 ±0.55	0.00 ±0.03	0.13 ±0.06	>18.43 ±2.05 *
PMVEMA	0.00 ±0.00	>40.55 ±6.59 *	0.00 ±0.00	0.32 ±0.10	1.04 ±0.09	0.02 ±0.08	>17.93 ±1.53 *	>19.50 ±4.10 *	>15.07 ±1.44 *	>12.08 ±3.21 *
PVP	0.03 ±0.03	7.28 ±0.55	0.02 ±0.03	>8.73 ±1.29 *	1.43 ±0.08	>20.50 ±14.42 *	>12.05 ±1.42 *	>12.60 ±1.57 *	>14.77 ±8.81 *	>24.37 ±0.80 *

^1^ Polymer concentration is expressed as mean ± standard deviation (*n* = 3) with units of % *w*/*v*. Concentrations represent polymer that was dissolved and/or possibly suspended as nano-sized particles. The definition and color scheme for solubility categories is provided in Table 1. * Sample appeared fully dissolved.

## Data Availability

Data is contained within the article or Supplementary Materials.

## Supplementary Materials

The following supporting information can be downloaded at https://www.mdpi.com/article/10.3390/pharmaceutics18060692/s1, Figure S1: TT stability during lyophilization and sonication; Figure S2: Representative images of lyophilized TT particles settling in 2-propanol with dissolved PEOX;T; Figure S3: Representative images of MTX particles settling in acetone with dissolved PAA; Figure S4: Representative images of insulin particles settling in ACN with dissolved PVP; Table S1: Summary of HPLC operating parameters for quantification of methotrexate and insulin; Figure S5: HPLC calibration curves; Figure S6: Representative HPLC chromatograms of methotrexate; Figure S7: Representative HPLC chromatograms of insulin (Method 1); Figure S8: Representative HPLC chromatograms of insulin (Method 2); Table S2: Relative polarities, boiling temperatures, and densities of organic solvents and water; Figure S9: Comparison of API particles before and after suspending and drying in organic solvents; Figure S10: Stability of MTX after casting and drying on PDMS chips; Figure S11: Stability of insulin after casting and drying on PDMS chips; Figure S12: Insulin microneedle patches.
