# Supplementary material for: Fabrication of Microneedle Patches by Suspension Casting of Drugs in Organic Solvents
Source: Pharmaceutics. 2026 Jun 1;18(6):692. doi: 10.3390/pharmaceutics18060692 (PMC13305238; doi:10.3390/pharmaceutics18060692)
Supplement: Supplementary file 1 [file pharmaceutics-18-00692-s001.zip › pharmaceutics-4291584-supplementary.pdf]

Supplementary Materials: Fabrication of Microneedle Patches by Suspension Casting of Drugs in Organic Solvents

Chao-Yi Lu, Lara Vaid, Asha Adler, Gulcin Arslan Azizoglu, Andrey V. Romanyuk and Mark R. Prausnitz

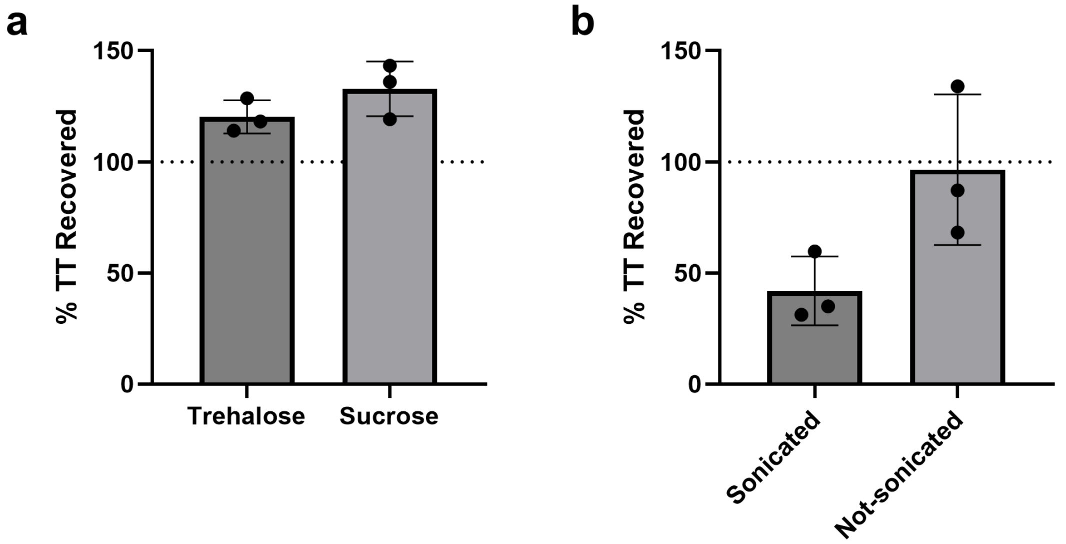

Figure S1. TT stability during lyophilization and sonication. (a) Percentage of antigenic TT recovered after lyophilization with trehalose or sucrose. TT was prepared by lyophilizing ~2.5 Lf TT with 5 mg trehalose or 5 mg sucrose in 92.8 µL deionized water. Similar settings were used for lyophilization as for the preparation of lyophilization of TT in the main text. (b) Percentage of antigenic TT retained after sonicating or manually mixing (not-sonicated) lyophilized TT particles suspended in chloroform. A lyophilized cake (~2.5 Lf TT in 5 mg sucrose) was sonicated for 1 s at the lowest amplitude setting (Vibra-Cell, Sonics & Materials, Newtown, CT, USA) in 400 µL chloroform or manually mixed with a metal spatula. The samples were dried at room temperature in a fume hood and reconstituted with PBST. TT antigenicity was determined by ELISA. Results are reported as mean ± standard deviation (n = 3).

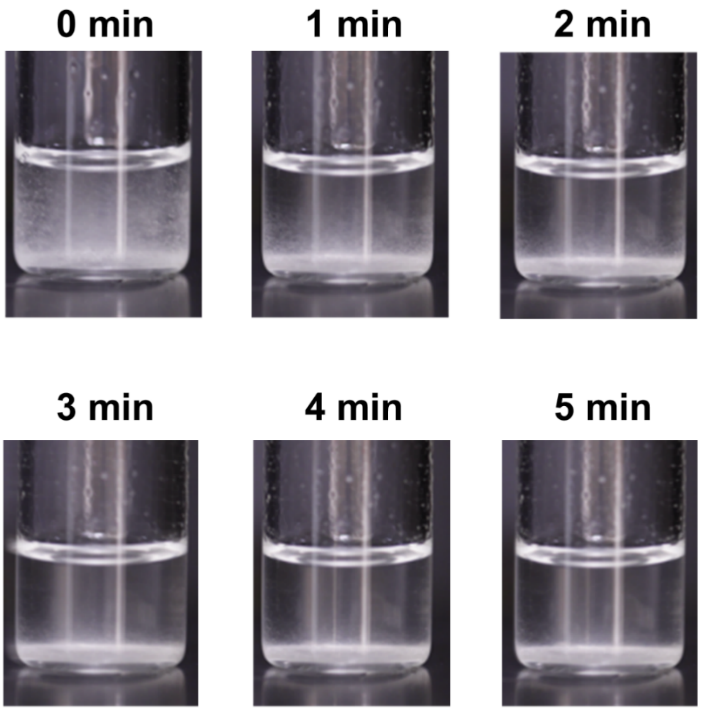

Figure S2. Representative images of lyophilized TT particles settling in 2-propanol with dissolved PEOX. Lyophilized TT (~50 Lf TT in 5 mg sucrose) was suspended in 800 μL of 2-propanol with 9.5% w/v dissolved PEOX. The mixture was homogenized (PowerGen 800, Fisher Scientific, Waltham, MA, USA) for 8 s just before starting the experiment.

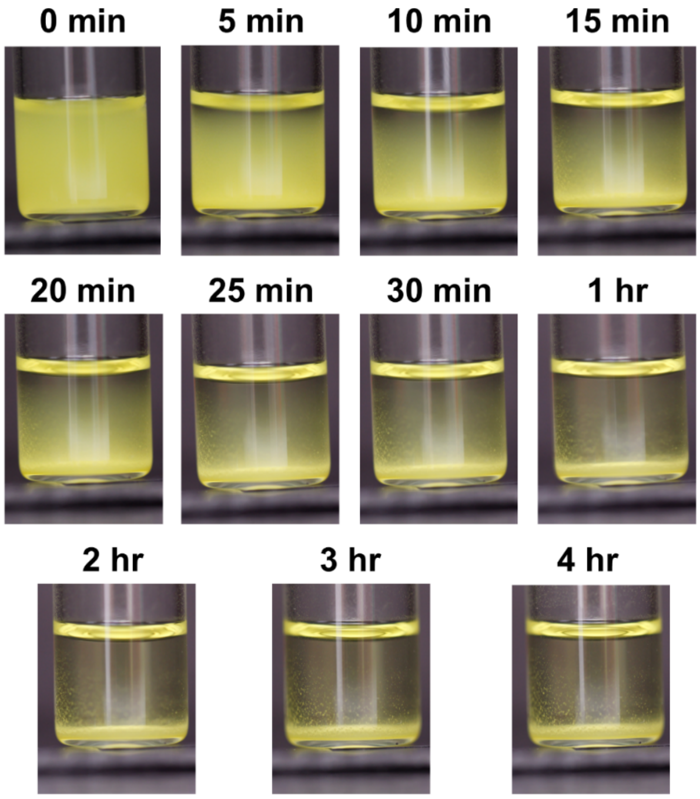

Figure S3. Representative images of MTX particles settling in acetone with dissolved PAA. MTX was suspended at a concentration of 1 mg/mL in acetone with 9% w/v dissolved PAA. The mixture was vortexed just before starting the experiment.

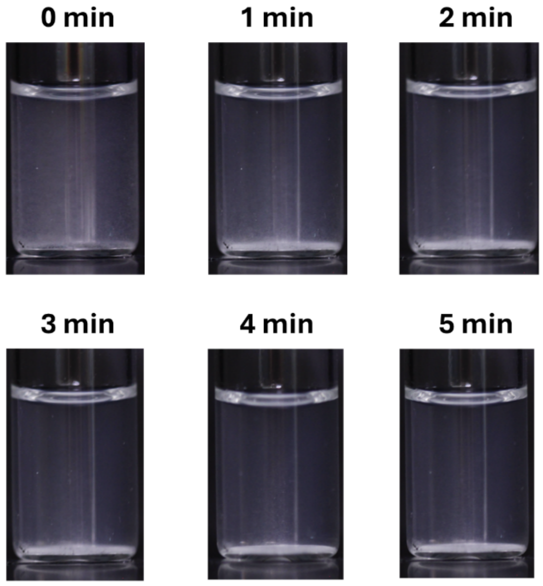

Figure S4. Representative images of insulin particles settling in ACN with dissolved PVP. Insulin was suspended at a concentration of 1 mg/mL ACN with 9% w/v dissolved PVP. The mixture was vortexed just before starting the experiment.

Table S1. Summary of HPLC operating parameters for quantification of methotrexate and insulin.[1]

API	Mobile Phase	Flow Rate (mL/min)	T (°C)	λ (nm)	RT (min)	Method time (min)
Methotrexate	ACN : 0.1% TFA in water (15:85)	1	30	303	4.5	10
Insulin 1	ACN : 0.1% TFA in water (30:70)	1	30	214	5.5	15
Insulin 2	ACN: 0.1% TFA in water (33:67)	0.8	30	232	6.5	20

[1]Abbreviations: acetonitrile (ACN), trifluoroacetic acid (TFA), UV detection wavelength (λ), column temperature (T), retention time (RT).

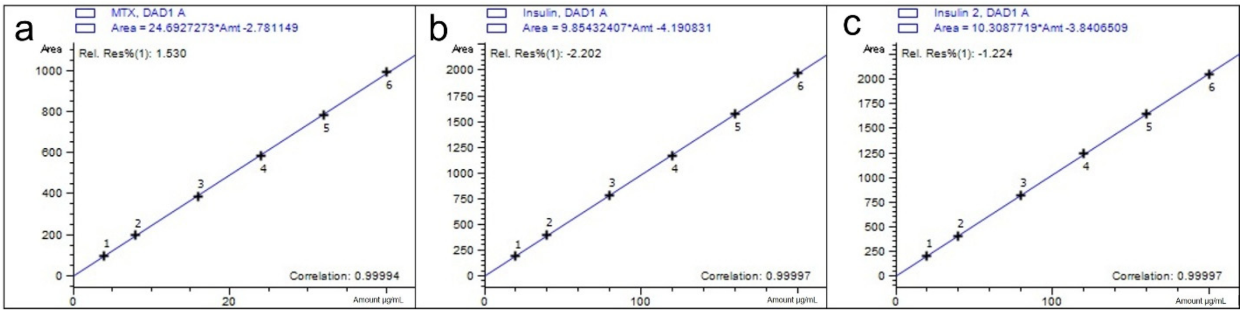

Figure S5. HPLC calibration curves. Standard linear calibration curves utilized for the quantification of active pharmaceutical ingredients (APIs) : (a) methotrexate (MTX) analyzed via a mobile phase of ACN : 0.1% TFA in water (15:85) at a flow rate of 1 mL/min , (b) insulin analyzed via Method 1 utilizing a mobile phase of ACN : 0.1% TFA in water (30:70) at a flow rate of 1 mL/min , and (c) insulin analyzed via Method 2 utilizing a mobile phase of ACN : 0.1% TFA in water (33:67) at a flow rate of 0.8 mL/min. The x-axis concentrations are expressed in µg/mL. Linearity and calibration accuracy across the operational ranges are demonstrated by correlation coefficients exceeding 0.9999 for all three methods.

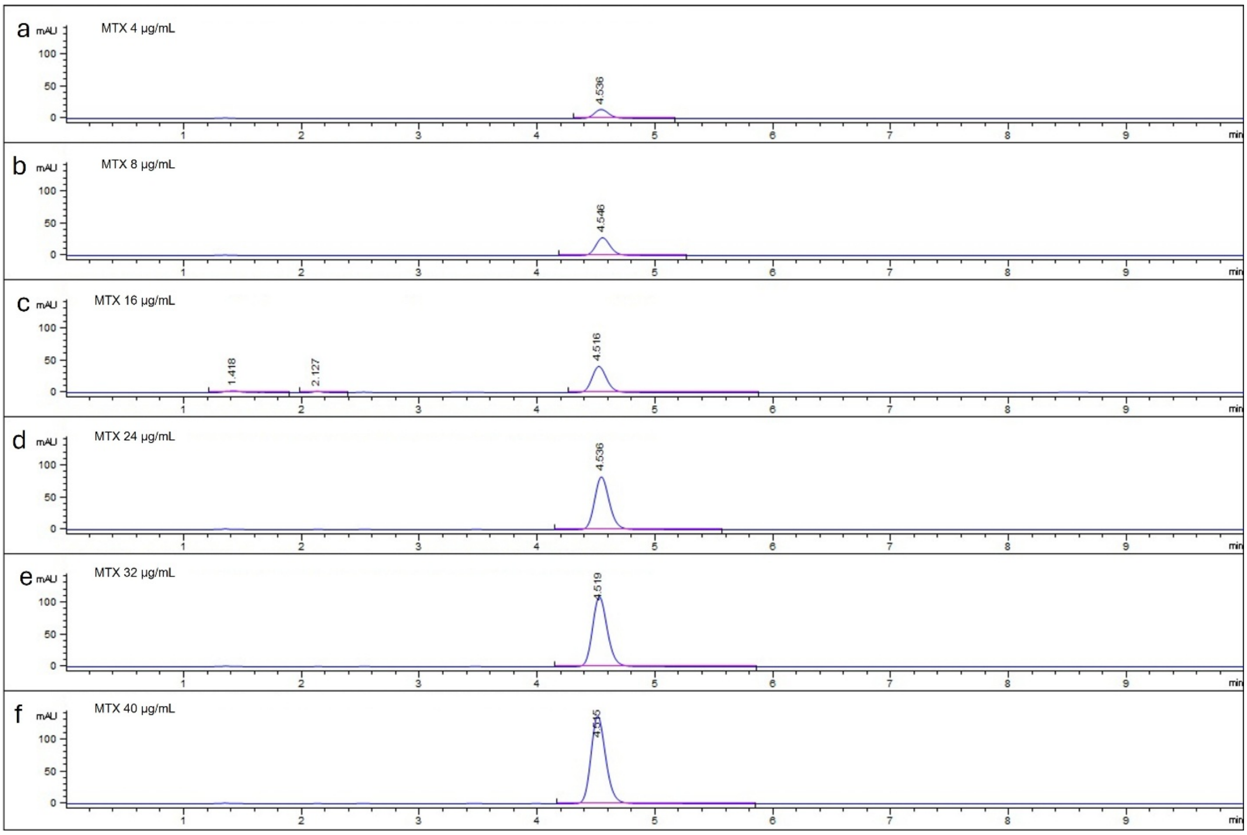

Figure S6. Representative HPLC chromatograms of methotrexate. Stacked chromatographic profiles of methotrexate (MTX) standard calibration standards across an operational concentration range of 4-40 µg/mL.

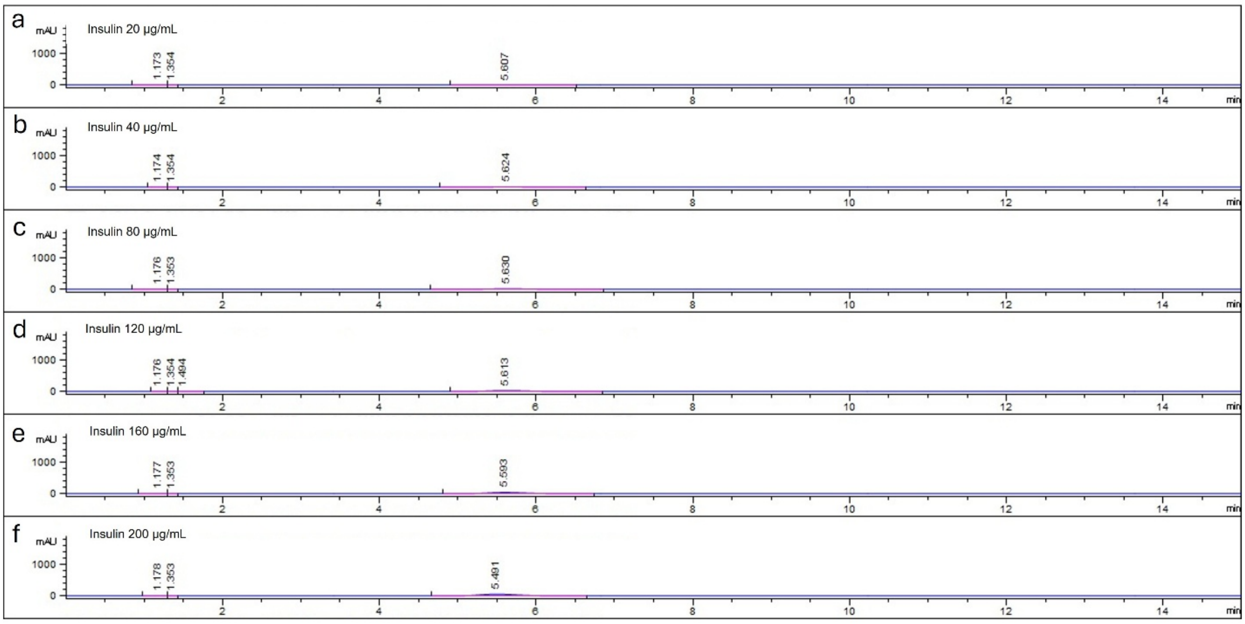

Figure S7. Representative HPLC chromatograms of insulin (Method 1). Stacked chromatographic profiles of insulin standard calibration concentrations ranging from 20-200 µg/mL analyzed via Method 1.

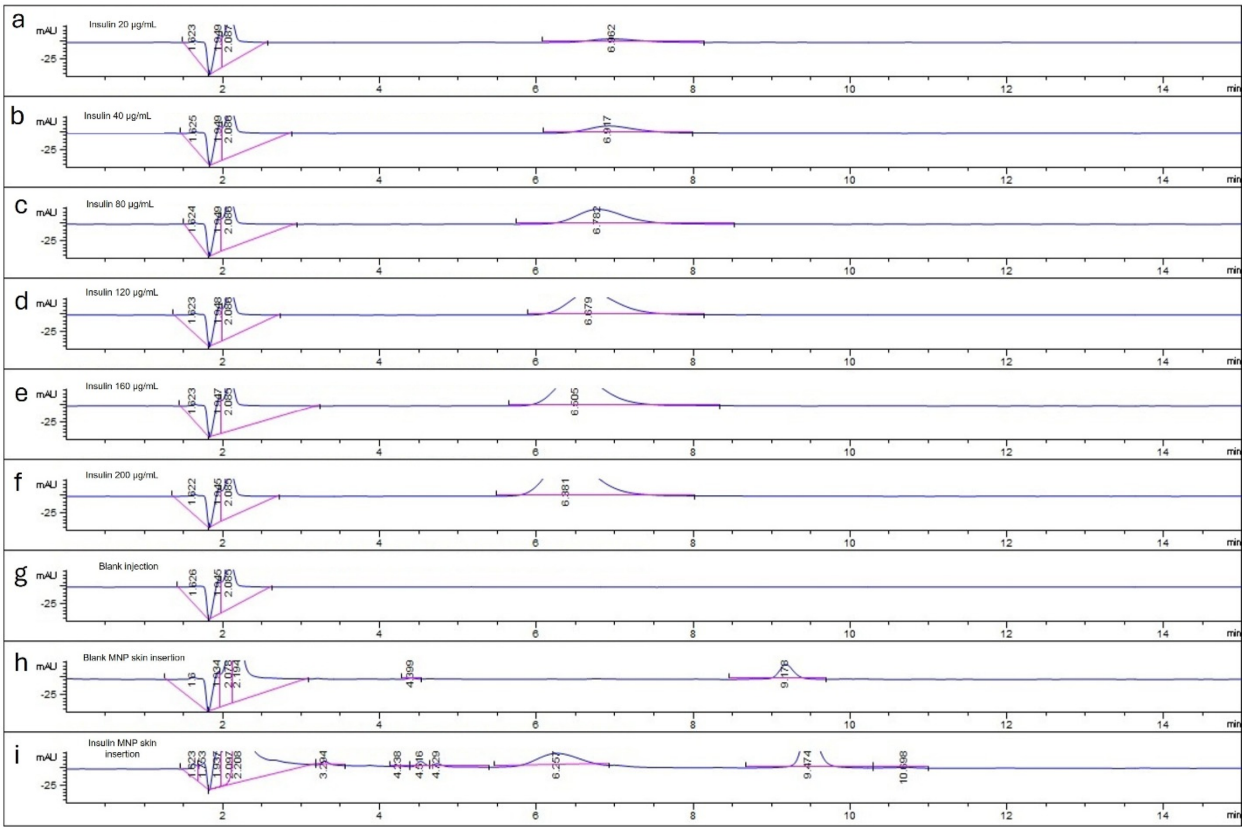

Figure S8. Representative HPLC chromatograms of insulin (Method 2). Stacked chromatographic profiles of insulin standard calibration concentrations and skin extraction matrices analyzed via Method 2. Chromatographic tracking profiles utilized for definitive microneedle patch (MNP) mass loading and delivery efficiency evaluations: (a–f) standard calibration injections at concentrations of 20-200 µg/mL, (g) blank solvent injection, (h) control extract of a drug-free PVP MNP applied to *ex vivo* porcine skin tissue, and (i) representative sample of an insulin-loaded PVP MNP extracted after *ex vivo* porcine skin insertion.

Table S2. Relative polarities, boiling temperatures, and densities of organic solvents and water[1,2].

Solvent	Relative Polarity[3]	Normal Boiling Temperature (°C)	Density (g/mL)
Toluene	0.099	110.6	0.867
Dioxane	0.164	101.1	1.033
Ethyl acetate	0.228	77	0.895
Chloroform	0.259	61.2	1.479
Acetone	0.355	56.2	0.785
Acetonitrile	0.460	81.6	0.786
2-Propanol	0.546	82.4	0.785
Ethanol	0.654	78.5	0.789
Methanol	0.762	64.6	0.791
Water	1.000	100	0.988

[1] Relative polarities and normal boiling temperatures were obtained from Murov, S. *Properties of Solvents Used in Organic Chemistry*. 2020; Available at: http://murov.info/orgsolvents.htm. Accessed on October 6, 2025. [2] Densities obtained from ACS Division of Organic Chemistry. *Common Solvents Used in Organic Chemistry: Table of Properties*. 2022; Available at: https://organicchemistrydata.org/solvents/. Accessed on December 1, 2025. [3] Data were normalized relative to water from measurements of solvent-based shifts of absorption spectra.

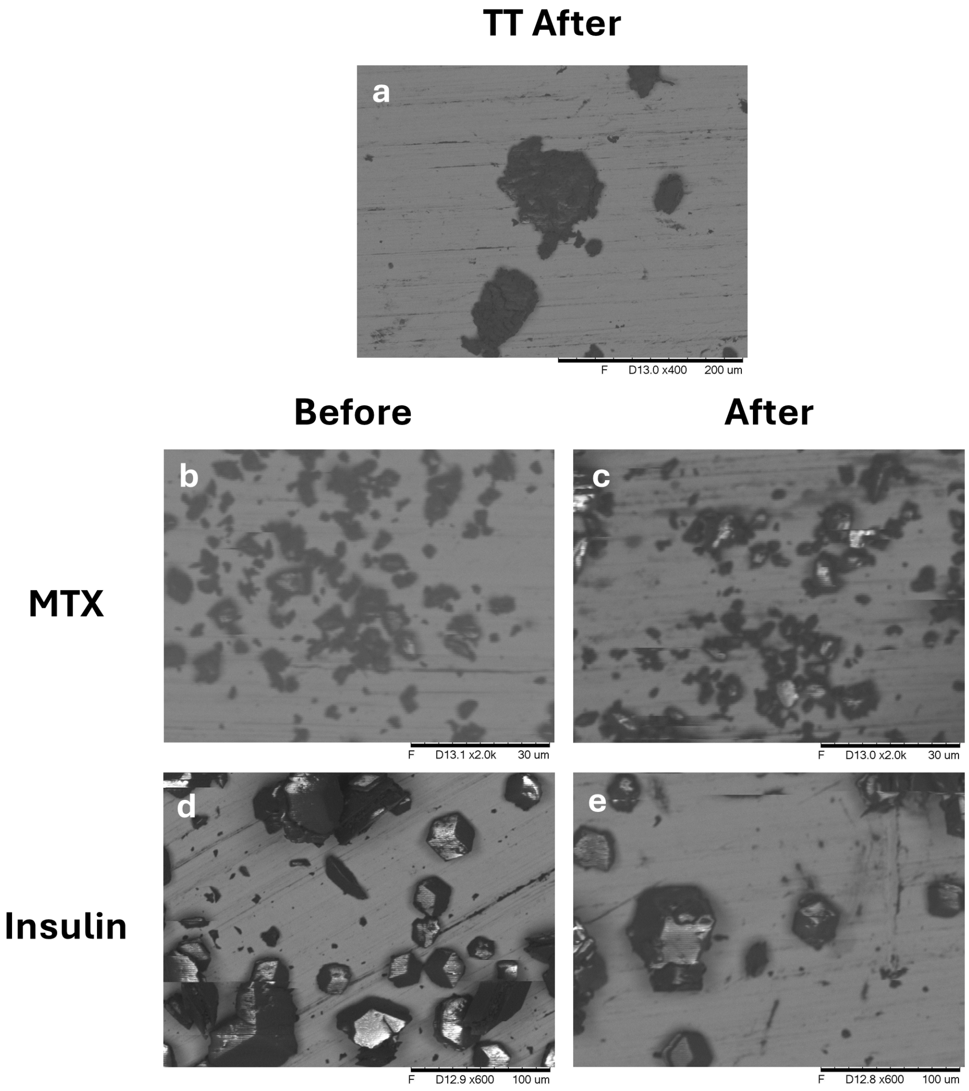

Figure S9. Comparison of API particles before and after suspending and drying in organic solvents. Representative SEM images of lyophilized TT particles (a) after drying, MTX particles (b) before and (c) after drying, and insulin particles (d) before and (e) after drying. Suspensions of each API were made by homogenizing lyophilized TT (~5 Lf TT with 5 mg sucrose) in 800 μL 2-propanol for 8 s, mixing 1 mg/mL MTX in acetone, and mixing 1 mg/mL insulin in ACN. MTX and insulin were dried at room temperature in a fume hood, and lyophilized TT was dried at room temperature in a vacuum oven operating at -95 kPa. Imaging was performed by SEM (TM3000, Hitachi High-Technologies Corporation, Tokyo, Japan).

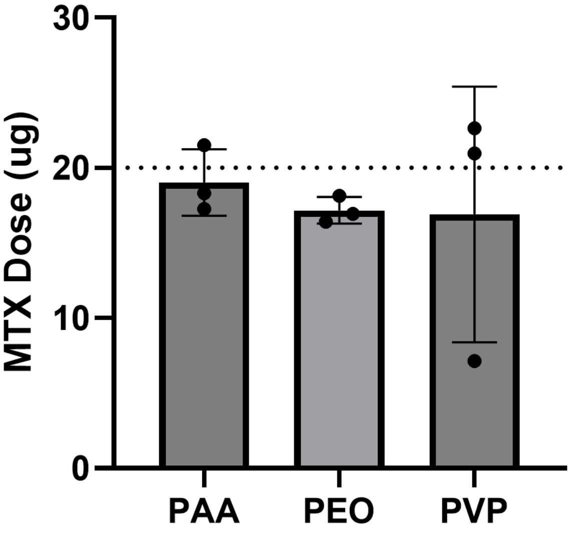

Figure S10. Stability of MTX after casting and drying on PDMS chips. MTX was suspended in acetone with 9% w/v dissolved PAA, ACN with 8% w/v dissolved PEO, or ACN with 9% w/v dissolved PVP. The casting solution contained 20 µg MTX (dashed line). Twenty microliters of each suspension were cast onto a PDMS chip and dried overnight in a chemical hood. The dried films were then reconstituted in 1 mL of 75% TFA-water and 25% ACN to determine the amount of MTX after drying by HPLC. Results are reported as mean ± standard deviation (n = 3). No significant differences were found among the three groups (one-way ANOVA, $p > 0.05$).

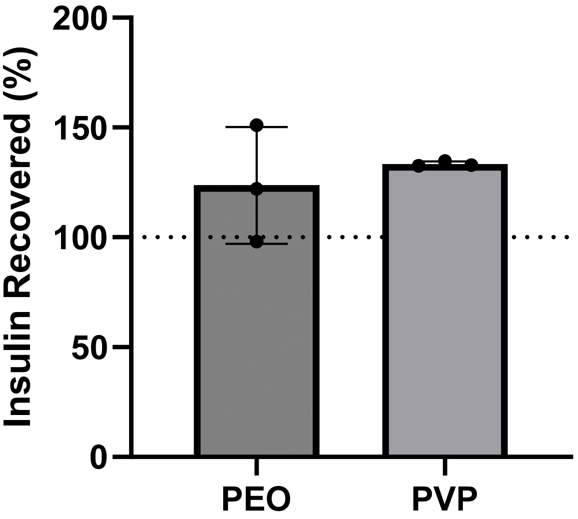

Figure S11. Stability of insulin after casting and drying on PDMS chips. Insulin was suspended in: ACN with 9.5% w/v dissolved PEO or ACN with 9.5% w/v dissolved PVP. After casting and drying on PDMS chips, the samples dried overnight in a chemical hood. The following day, the dried films were reconstituted in 70% TFA-water and 30% ACN, and analyzed by HPLC. Results are reported as mean ± standard deviation (n = 3). An unpaired t-test showed no significant difference between the two groups ($p > 0.05$).

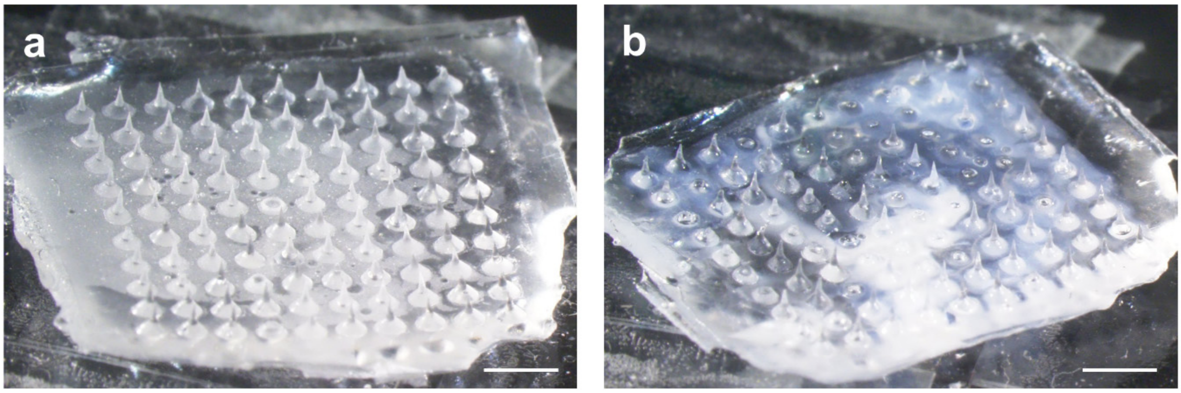

Figure S12. Insulin microneedle patches. Representative microscopic images of an insulin MNP (a) before and (b) after reconstitution with TFA-water. To make the MNPs, 60 μL of 1 mg/mL insulin suspended in 9.5% w/v PEO dissolved in ACN was cast onto a PDMS mold. After drying, an epoxy backing was applied onto the mold. The following day, the MNP was demolded and reconstituted in TFA-water for ~1 min. Scale bar = 2 mm.
